# Early Divergent Host Responses in SHIVsf162P3 and SIVmac251 Infected Macaques Correlate with Control of Viremia

**DOI:** 10.1371/journal.pone.0017965

**Published:** 2011-03-25

**Authors:** Huanbin Xu, Xiaolei Wang, Lisa A. Morici, Bapi Pahar, Ronald S. Veazey

**Affiliations:** 1 Division of Comparative Pathology, Tulane National Primate Research Center, Covington, Louisiana, United States of America; 2 Department of Microbiology and Immunology, Tulane University School of Medicine, New Orleans, Louisiana, United States of America; Statens Serum Institute, Denmark

## Abstract

We previously showed intravaginal inoculation with SHIVsf162p3 results in transient viremia followed by undetectable viremia in most macaques, and some displayed subsequent immunity to superinfection with pathogenic SIVmac251. Here we compare early T cell activation, proliferation, and plasma cytokine/chemokine responses in macaques intravaginally infected with either SHIVsf162p3 or SIVmac251 to determine whether distinct differences in host responses may be associated with early viral containment. The data show SIVmac251 infection results in significantly higher levels of T cell activation, proliferation, and a mixed cytokine/chemokine “storm” in plasma in primary infection, whereas infection with SHIVsf162p3 resulted in significantly lower levels of T cell activation, proliferation, and better preservation of memory CD4+ T cells in early infection which immediately preceded control of viremia. These results support the hypothesis that early systemic immune activation, T cell proliferation, and a more prominent and broader array of cytokine/chemokine responses facilitate SIV replication, and may play a key role in persistence of infection, and the progression to AIDS. In contrast, immune unresponsiveness may be associated with eventual clearance of virus, a concept that may have key significance for therapy and vaccine design.

## Introduction

Rhesus macaques are the premier animal model for studying the pathogenesis and immunology of HIV infection. However, there are numerous simian immunodeficiency viruses (SIVs) and chimeric simian/human immunodeficiency viruses (SHIVs), each with different characteristics and pathogenicity. Most SHIVs are easily transmissible and initially highly infectious, yet most SHIV-infected macaques fail to maintain plasma viremia for more than a few weeks/months, making these viruses less useful for pathogenesis or vaccine studies. For example, SHIVsf162P3 was originally described as a pathogenic SHIV, and some rhesus macaques clearly develop AIDS when infected with this virus [1], yet while most SHIV-infected macaques have marked peak viremia, and often similar to those infected with more pathogenic viruses, the majority fail to sustain viremia, and most clear infection, at least to undetectable levels in plasma within a few weeks of infection. Although the reasons for these disparate outcomes are unknown, few studies have directly compared the host responses in identical cohorts of macaques inoculated with a pathogenic virus (SIVmac251) with those infected with a virus that results in early high viremia, but is eventually cleared to undetectable levels in plasma, such as SHIVsf162P3.

In macaques, SIVmac251 is among the most widely used virus for examining the immunology and pathogenesis of HIV infection in humans, largely due to its high infectivity, persistent and stable post peak (set point) viremia, and high rate of progression to AIDS within 1–3 years. In contrast, the majority of SHIVsf162P3-infected macaques initially have high viremia, similar to animals infected with SIVmac251, yet most have undetectable viremia (<125 viral copies/ml) within 60–90 days of infection [2]. The fact that animals are easily infected, support high peak viral replication, and initially lose intestinal CD4+ T cells [3] demonstrate these animals are not inherently resistant to infection, and have ample viral target cells to support replication. Since most SHIV-infected animals “clear” or control infection to undetectable levels, we hypothesized there would be detectable differences in immune responses between macaques infected with SIV mac251, which may yield correlates of a protective immune response that prevents animals from progressing to AIDS.

Human immunodeficiency virus (HIV) infection in humans and experimental SIV infection in rhesus macaques results in peak viremia, marked non-specific immune activation, T cell proliferation, intestinal CD4+ T cell depletion, and in the chronic stage, persistently high viremia with eventual progression to AIDS [4], [5]. In contrast, natural primate hosts for SIV (such as African green monkeys, sooty mangabeys, etc.) generally do not progress to AIDS when infected with SIV, and maintain normal levels of T-cell activation, proliferation and apoptosis during chronic infection, despite persistently high levels of viral replication throughout infection [6], [7], [8], [9], [10]. Although early host responses against HIV and SIV are sometimes predictive of disease outcome [9], [11], [12], few correlates of immune protection or viral control have been identified to date. Perhaps this is because investigators have sought enhanced or more prominent immune responses associated with control of viremia. It has long been known that SIV and HIV selectively replicate in activated memory CD4+ T cells. More recently, abundant evidence indicates increased immune activation and T cell proliferation is a major correlate of increased viral replication and progression to AIDS in HIV and SIV infection [10], [13]. Thus, we compared these parameters in rhesus macaques inoculated with viruses that result in different outcomes, now hypothesizing that increased immune activation may actually be detrimental to the host, and that dampened immune responses may be associated with the clearance of virus from hosts infected with SHIVsf162p3. Examining potentially divergent immune responses between pathogenic and nonpathogenic infection with closely related viruses may yield clues regarding the pathogenesis of AIDS as well as the immune mechanisms involved in control of viremia. Here we compared levels of immune activation, cell proliferation, and cytokine/chemokine production in macaques infected with SHIVsf162p3 to those with SIVmac251.

## Materials and Methods

### Animals and virus infection

A total of thirty-six Indian-origin rhesus macaques (*Macaca mulatta*), which were initially negative for HIV-2, SIV, type D retrovirus, and STLV-1 infection were examined in this study. All animals were housed at the Tulane National Primate Research Center in accordance with the standards of the Association for Assessment and Accreditation of Laboratory Animal Care International. All studies were reviewed and approved by the Tulane University Institutional Animal Care and Use Committee. Of these, sixteen and fourteen SIV-negative animals were intravaginally inoculated with SHIVsf162P3 and SIVmac251 respectively as previously described and blood from another 6 uninfected animals were added to controls [14]. Experimental macaques were intramuscularly treated with Depo provera® (30 mg) 28–32 days prior to vaginal challenge with 300TCID_50_ of either virus which corresponded to 2.8×10^7^ RNA copies/ml of SIVmac251 and 9.1×10^7^ RNA copies of SHIVsf162p3 per inoculum.

### Sample collection and flow cytometry

Fresh blood was collected in EDTA Vacutainer tubes. Plasma was collected by centrifugation at 1000 g for 20 min at 4°C, aliquoted, and stored at −70°C until analysis.

Directly conjugated anti-human antibodies were obtained from BD Biosciences (San Jose, CA) unless stated otherwise. Peripheral blood was stained using a whole blood lysis protocol as previously described [14]. In brief, 100 µl whole blood was incubated with monoclonal antibodies for 30 min at 4°C, followed by a 7-min lyse with FACS lysing solution (Becton Dickinson, San Jose, Calif.). Cells were then washed (400×*g*, 7 min) and resuspended in 2% paraformaldehyde. Antibodies included anti-CD3 (clone SP34-2)-PE-Cy7 or Pacific Blue; anti-CD4 (clone L200)-Amcyan; anti-CD8 (3B5, Caltag)-PE-TxR or –Qdot 655; anti-HLA-DR (clone Immu-357, Beckman Coulter)–ECD; anti-CD95 (clone DX2)-PE-Cy5 and anti-CD28 (clone CD28.2, Biolegend)-Alexa 700. For assessing proliferation, whole blood was surface stained, treated with FACS lysing solution, washed, and intracellularly stained with anti-Ki67 (Clone B56) as previously described [15]. Samples were acquired on a FACSAria or LSR II (BD Biosciences) and data analyzed using Flow Jo software (Tree Star, Inc., San Carlos, CA).

### Viral quantification in plasma

Plasma viremia was quantified using a bDNA assay (Seimens Diagnostics Inc.) as previously described [16]. The sensitivity of detection was 125 viral RNA copies/ml plasma.

### Detection of cytokines/chemokines in plasma by multiplex microbead immunoassay

A multiplex biometric immunoassay, containing fluorescent dyed microspheres conjugated with a monoclonal antibody specific for the target protein was used for cytokine measurements according to the manufacturer's instructions (Bio-Plex Human Cytokine Assay; Bio-Rad Inc., Hercules, CA, USA). 27 cytokines were measured in the kit (171A11127-27 plex, Bio-Rad). Briefly, 50 µl diluted plasma (1∶1) was incubated with antibody-coupled beads. Complexes were washed, incubated with biotinylated detection antibody, and then streptavidin-PE. A range of 1.95–32,000 pg/ml of recombinant human cytokines was used to establish standard curves and maximize sensitivity of the assay. Cytokine levels were determined using a multiplex array reader from Luminex™ Instrumentation System (Bio-Plex Workstation from Bio-Rad Laboratories). Analyte concentrations were calculated using software provided by the manufacturer (Bio-Plex Manager Software).

### Statistics

Graphical presentation and statistical analysis of the data were performed using GraphPad Prism 4.0 (GraphPad Software, SanDiego, CA). Comparisons between groups or between time points were performed using a two-tailed unpaired and paired t-test. P values<0.05 were considered statistically significant.

## Results

### Differential replication of SHIVsf162P3 and SIVmac251 in acute infection of rhesus macaques

**Figure 1:**
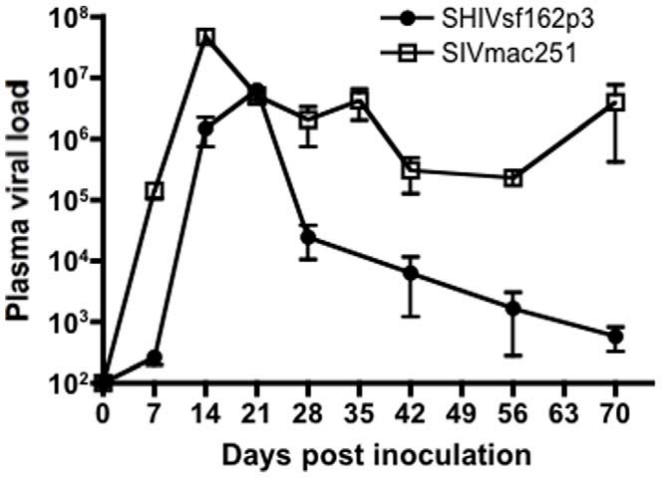
Plasma viremia in early SHIVsf162p3 (n = 11; filled circles) and SIVmac251 (n = 16; open squares) infected rhesus macaques. Means ±SEM are shown.

Here, we compared responses of macaques intravaginally inoculated with either SHIVsf162P3 or SIVmac251. As shown in **Fig. 1**, peak viral loads were slightly lower, and peak viremia occurred 1 week later in macaques infected with SHIVsf162P3 compared to SIVmac251, (**Fig. 1**). Mean peak viral loads ranged from 1.5×10^6^ to 6.3×10^6^ copies/ml in SHIVsf162p3-infected animals, and 5.1×10^6^ to 4.7×10^7^ copies/ml in SIVmac251-infected macaques at 14 and 21 days postinfection respectively. By 28 days of infection however, viral loads in SHIVsf162P3 infected animals were dramatically lower, and continually decreased to low or undetectable levels by 70 days of infection. In contrast, viremia remained high throughout infection in SIVmac251-infected animals (**Fig. 1**).

### Limited T-cell activation in early SHIV-infection

**Figure 2:**
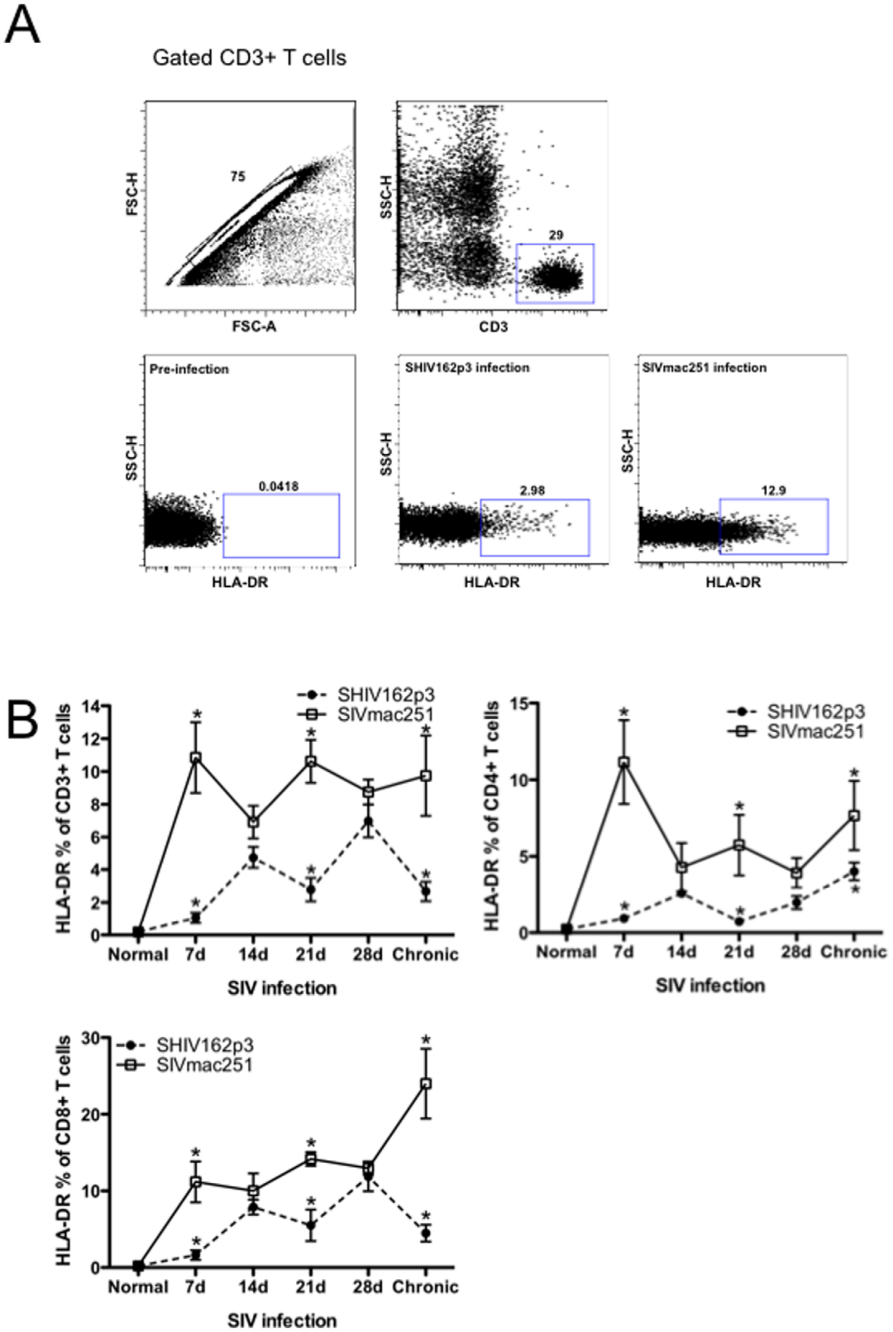
Dynamics of activated T cells in blood as determined by HLA-DR expression on T cell subsets. A) Representative gating strategy showing HLA-DR expression on CD3+ T cells prior to infection and 21 days after infection. B) Mean percentages of HLA-DR expression on total T cells (CD3+), and on CD4+ and CD8+ T cell subsets in SHIVsf162p3 (filled circles) and SIVmac251-infected (open squares) RMs in early and chronic (3 months) infection. Means±SEM are shown. Asterisks indicate significant differences (P<0.05) between groups.

To characterize early host responses, HLA-DR expression was examined on T cells to assess levels of activation after infection. The representative gating strategy for HLA-DR+ T-cell is shown in Figure 2A). Throughout acute infection, percentages of activated (HLA-DR+) CD3+, CD4+ and CD8+ T cells in SHIV162p3-infected animals were consistently lower in macaques infected with SHIV than SIVmac251. Levels of T cell activation were already significantly higher in SIVmac251-infected macaques at 7 days (p<0.001 for CD3+; p<0.001 for CD4+ and p<0.05 for CD8+ T cells) and 21 days post infection (p<0.01 for CD3+; p<0.05 for both CD4+ and CD8+ T cells) (**Fig. 2**). Although levels seemed to converge by 28 days, HLA-DR expression was consistently and persistently higher in SIVmac infected than SHIV infected macaques, and significantly higher in chronic infection (Fig. 2). These data demonstrate distinct immunologic sequela apparent in very early infection that may distinguish immune responses in macaques that sustain plasma viremia from those that eventually control viral replication.

### Correlation of early T cell proliferation and peak SIV viremia

**Figure 3:**
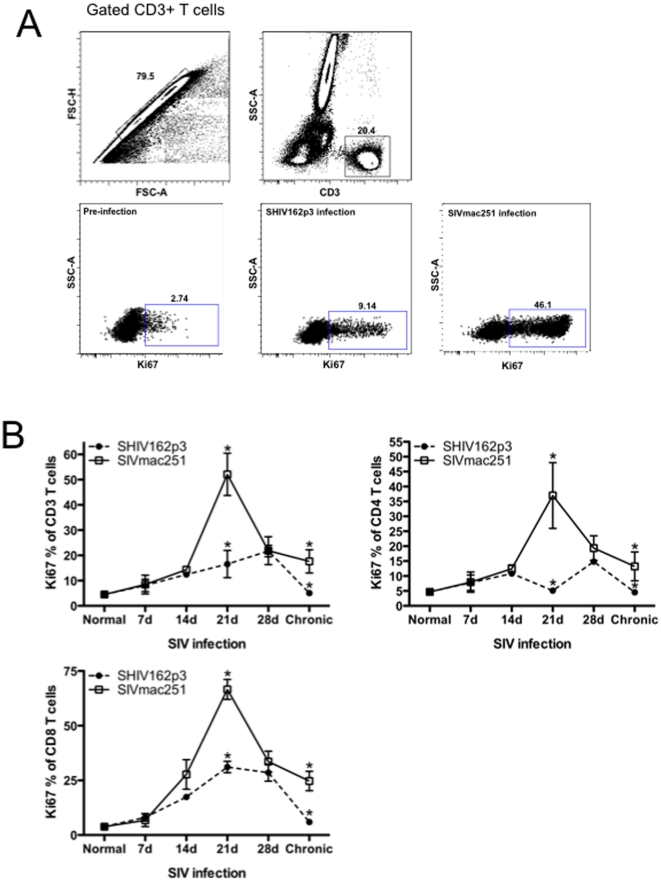
T-cell proliferation during acute SIV infection. A) Representative gating strategy showing Ki-67 expression on CD3+ T cells prior to and 21 days after infection. B) Longitudinal analysis of early T-cell proliferation in blood from SHIVsf162p3 (filled circles) and SIVmac251-infected RMs (open squares). Means±SEM are shown. Significant differences (P<0.05) between groups are indicated by asterisks.

Recent studies suggest that the kinetics of proliferating T and B cells may be related to the dynamics of early SIV replication in progressing and nonprogressing hosts [17], [18]. Here we compared T-cell proliferation using Ki-67 as a marker of cycling lymphocytes in acute SHIVsf162P3 and SIVmac251-infection. The representative gating strategy for Ki67+ T-cell is shown in Figure 3A). Peak T cell proliferation occurred by 21 days postinfection in SIVmac251 infected macaques, and CD4+ T cell proliferation increased significantly from 14 to 21 days (from 3.427% to 19.37%) (**Fig. 3**). At 21 days of infection, the level of proliferating CD3+T cells, CD4+T cells and CD8+T cells in SIVmac251 infected macaques were all significantly higher than those infected with SHIV162p3 (from 16.56% to 56.1%, 5.096% to 37%, 31.12% to 66.58% (p<0.001; p<0.05 and p<0.01 respectively), However T-cell proliferation decreased thereafter, and by day 28, values were indistinguishable between cohorts. However, T cell proliferation continued to diminish in SHIV162p3-infected animals to baseline levels, but stabilized in SIVmac251 infected animals at levels significantly higher than those of baseline, or SHIV-infected animals, indicating sustained T cell proliferation/activation in SIVmac infection.

### Comparison of early cytokine/chemokine responses in acute SHIV and SIV infection

**Figure 4:**
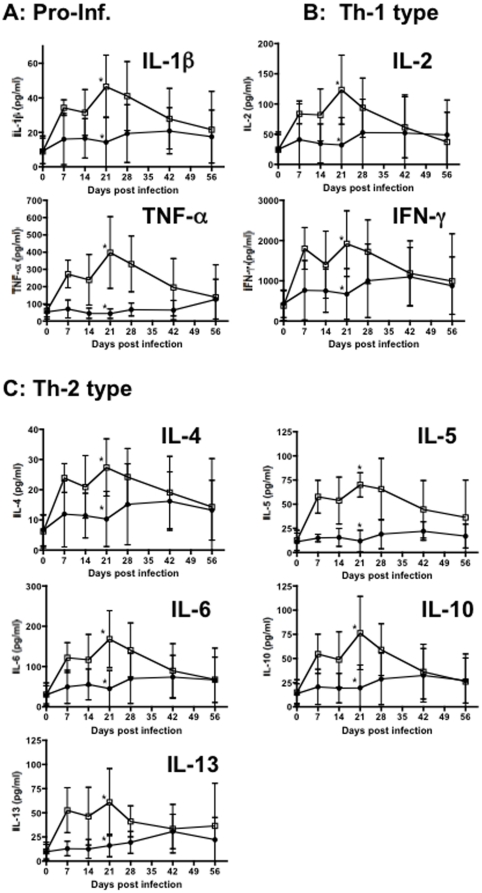
Levels of proinflammatory cytokines (A), Th1 cytokines (B), and Th2 cytokines (C) in plasma of SHIVsf162p3 (filled circles) and SIVmac251-infected RMs (open squares). Means±SEM are shown. *Asterisks indicate significant differences between groups (P<0.05).

As described above and illustrated in **Figs. 2** **–** **3**, pathogenic SIVmac251 infection results in marked immune activation and cell proliferation that is likely predictive of disease progression. Since lymphokines play a key role in immune regulation, this activation/proliferation should be accompanied by burst of cytokine/chemokine release upon SIV infection. To further correlate early host immune responses with immune activation and viremia, a comprehensive quantitative analysis of lymphokines produced in response to SHIVsf162P3 and SIVmac251-infection in macaques was performed. As shown in **Fig. 4A**, pro-inflammatory cytokines including IL-1β and TNF-α were markedly elevated and sustained in plasma of SIV infected macaques thorough 21 days of infection. In contrast, levels of these cytokines were markedly lower in macaques infected with SHIV162p3 at this time point (P<0.05). In HIV/SIV infection, an imbalance of Th-1 Th2 cytokines has been suggested to occur. However, we found that in both SHIV162p3 and SIVmac251 infected macaques, both Th-1 (IL-2, IFN-γ) and Th-2 (IL-4, IL-5, IL-6, IL-10, and IL-13) type cytokines were markedly elevated after infection. Further, levels of all cytokines were significantly higher in macaques infected with SIVmac than in SHIV162p3 at day 21 (P<0.05) (**Fig. 4B, C**). Other cytokines such as IL-7, IL-9 and IL-17 were markedly higher in SIVmac251-infected macaques than in SHIVsf162P3 infected animals (data not shown). Combined, these data indicate that pathogenic SIVmac251 infection results in much stronger early immune responses including increased cytokine secretion, T cell activation, and proliferation, and this early response may correlate with a poor prognosis.

**Figure 5:**
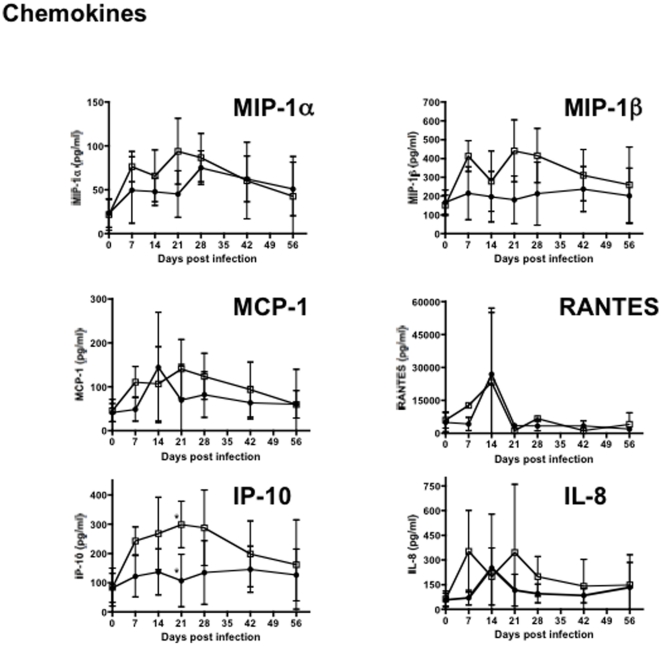
Changes in chemokine levels in plasma in acute SHIVsf162p3 (filled circles) and SIVmac251-infected RMs (open squares). Means±SEM are shown. *Asterisks indicate significant differences between groups (P<0.05).

In addition to cytokines, levels of several chemokines including MIP-1α, MIP-1β, MCP-1, RANTES, IL-8 and IP-10 were also elevated in plasma of animals infected with either virus (**Fig. 5**). Both infections resulted in increased levels of chemokines in plasma, but only IP-10 showed significant differences between SHIVsf162P3 and SIVmac251 inoculated macaques (**Fig. 5**). IP-10 expression was significantly up-regulated in SIVmac251 infection at day 21, compared with SHIVsf162P3 infection (P<0.05). As an IFN-inducible protein, increased IP-10 expression further indicates that SIVmac251 inoculation results in immune activation and a brief, yet marked cytokine/chemokine “storm” that is not paralleled in intensity by less pathogenic infections, despite the similar high primary viremia in both cohorts.

These results demonstrate pathogenic SIV infection results in a marked cytokine/chemokine inflammatory response during acute infection, which likely promotes ongoing viral replication, and may mediate immunopathology by favoring both direct and bystander immune activation, T cell proliferation, and may predict disease progression. Interestingly however, lymphokine levels essentially converge in macaques infected with either virus by day 42, regardless of the inoculum/infection. Elevated plasma levels of cytokines in SIVmac251 infected animals provide additional evidence that immune activation is associated with pathogenic infection. Further, and similar to SIV infection of natural or non-progressing hosts [7], [8], [19] SHIV162p3 infected macaques had a relatively quiescent lymphokine response, as most cytokines/chemokines hardly changed from baseline levels throughout primary infection, despite high viremia at 14–28 days of infection. Note that IL-1, IL-6, IL-10, IL-12, TNF-α and IFN-γ, levels in SIVmac251-infected animals were significantly higher than SHIV162p3 infected macaques at 21 days postinfection (P<0.05) which was peak viremia for SHIV162p3-infected macaques. In summary, pathogenic SIVmac251 infection results in significantly higher levels of essentially all major classes of cytokines as well as several chemokines compared to SHIVsf162P3 infection. Although a definitive cause and effect relationship was beyond the scope of this study, higher levels of lymphokines in plasma clearly correlate with increased cell activation and T cell proliferation, which may contribute to the sustained viremia in SIVmac251 infected cohorts.

### Equal loss of peripheral central memory CD4+ T cells in early infection

**Figure 6:**
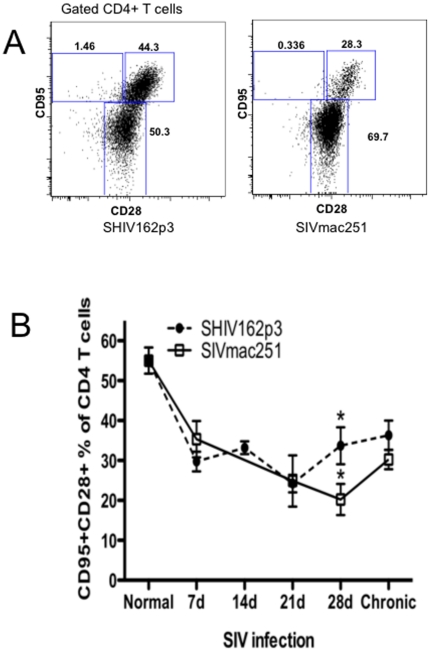
Comparison of central memory CD4+ T cells in SIV (open squares) and SHIVsf162p3 (filled circles) infected macaques. A) Representative FACS dot plot of central memory CD4+T cells (CD28+CD95 gated CD4+ T cells) in blood at 28 days after SHIVsf162p3/SIVmac251 infection. B, Dynamic of central memory CD4+ T cells in peripheral blood of SIVmac251 (open squares) versus SHIVsf162P3 (filled circles) infected macaques. Although percentages are almost identical in early infection, significantly higher percentages of central memory CD4+ T cells recover in SHIVsf162p3-infected macaques by 28 days of infection. Means ± SEM are shown. *Indicates significant differences between groups (P<0.05).

As shown above and in previous studies, macaques intravaginally infected with SHIV162p3 generally do not progress to AIDS, and survival has been associated with preservation or restoration of central memory CD4+ T lymphocytes. Thus, we compared changes in blood of central memory CD4+ T cells in early SHIV162p3 and SIVmac251 inoculation. The representative gating strategy for central memory CD4+ T-cells is shown in Fig. 6A). Remarkably, and despite significant differences in immune activation and lymphocike production, percentages of peripheral CD4+ central memory T cells were markedly and reduced in both viruses in early infection, with no apparent differences detected before 21 days p.i between groups (**Fig. 6B**). However, percentages of central memory CD4+ T cells then increased in SHIV162P3-infected macaques at day 28 postinfection, to levels higher than SIVmac251-infected animals (from 33.7% to 20.23%, P<0.05). In chronic phase (3 month p.i.), the frequency of circulating central memory CD4+ T-cells in SIVmac251-infected animals increased to some extent, and there were no significant differences compared with SHIV162p3 infected animals, although levels remained higher than in SIVmac251 infected macaques. Combined, these data suggest that in early infection, viral replication and target cell infection and destruction are similar in the two infections, yet eventually, viremia is controlled and central memory CD4+ T cells begin to recover in SHIV-infected macaques.

## Discussion

We previously reported that intravaginal inoculation with SHIV162p3 results in transient viremia followed by undetectable viral loads in most macaques, yet many of these animals showed remarkable protection to subsequent intravenous SIVmac251 challenge. Although the prior study clearly showed prior infection confers resistance to superinfection/viremia with a more pathogenic virus, the mechanisms of this protection remain unclear.

The current study demonstrated that intravaginal inoculation of naïve RMs with either SHIVsf162P3 or pathogenic SIVmac251 results in early active viral replication, T-cell activation, proliferation, and a burst of cytokine/chemokine release, but SIVmac251 infection results in significantly higher levels of activation and lymphokine production very early in infection compared to SHIVsf162P3 infection. Since the level of central memory CD4+ T cell loss was similar at these timepoints, this suggest that the early immune response to HIV-1 infection is likely to be an important factor in determining the clinical course of disease. Further, it has been proposed that very early impairment of immune responses may contribute to subsequent viral escape mutations [20]. Combined, these data all suggest very early divergence in immune responses to SIV infection could be predictive of disease outcome and vaccine efficiency.

Resistance to progressive or pathogenic infection in SIV/SHIV infected macaques may be associated with an effective host immune response, as some individuals maintain high viremia and progress to AIDS, whereas most eventually clear infection and are resistant to subsequent challenge. However, unlike macaques, primate species that naturally resist disease progression when infected with SIV (African Green monkeys, Sooty mangabeys, etc) still harbor high viremia, despite lack of progression to AIDS. Although these animals have evolutionary adaptations that are likely responsible for lack of disease progression, infection of natural hosts is also characterized by limited activation, proliferation, and preserved central memory T cells [7], [8], [19]. Here we compared early host responses within identical cohorts of rhesus macaques following intravaginal SHIVsf162p3 and SIVmac251 inoculation. These data show the early dynamics of T-cell activation, proliferation and cytokine levels in plasma positively correlated with virus replication. Immune responses for almost all parameters tested were significantly higher in SIVmac251 than SHIVsf162P3-infected macaques. Of note, host responses gradually converged to similar levels in both cohorts by 28 days of infection, which suggests early host responses such as levels of T-cell activation and proliferation are key to disease progression, and early suppression may be key to viral containment, a theory previously proposed for non-progressing host species.

Higher immune activation and proliferation after SIV infection is purportedly associated with higher levels of T cell apoptosis, and central memory CD4+ T-cells are considered preferential targets for “bystander” activation [21], [22]. However, the level of central memory CD4+ T cell destruction was essentially identical in early infection, indicating that target cell availability of ability to infect/destroy these cells was not likely a factor in early infection or the outcome.

Importantly, the early cytokine responses were largely dominated by the induction of proinflammatory cytokines, which may amplify the immunopathology during early HIV infection [11], [23]. In pathogenic SIVmac251 infection, marked increases in essentially all cytokines/chemokines tested were detected in plasma through 56 days postinfection, and significantly elevated levels of IL-1β, IL-6, IL-10 and TNF-α, were detected at day 21 infection in SIVmac251-infected macaques compared with SHIV162P3 infected macaques. Elevated levels of several cytokines/chemokines in plasma are consistent with marked immune activation (a cytokine “storm”) which could result in increased viral infection and amplification in multiple cells and tissue sites. Further, this elevated cytokine milieu could favor continued or sustained immune cell activation, resulting in continued cell turnover, and destruction of viral target cells in SIV infection. Thus, early, *constrained* immune activation and proliferation might contribute to reduced lymphocyte destruction, preservation of memory T cells, and lower viral amplification in tissues, at least until effective immune responses can be mounted against potentially pathogenic infections.

In summary, marked differences in early host responses including T-cell activation and proliferation, and cytokine/chemokine responses were detected between macaques intravaginally infected with the minimally pathogenic SHIVsf162P3 and highly pathogenic SIVmac251, suggesting that early immune activation and proliferation are key factors in AIDS pathogenesis, and associated with disease outcome. Classically, investigators have sought enhanced immune responses for correlates of protection, and vaccine candidates are usually selected and advanced based on their ability to induce more potent immune responses to viral antigens. In contrast, in non-progressing host species, it has been proposed that suppressed immune response are associated with viral control. However, here we show that even within the same species of susceptible macaques, dampened immune responses to a virus that initially proliferates well within the host, may be associated with long term control and potential clearance of the virus. If so, perhaps vaccine strategies aimed at inducing temporary “tolerance”, rather than “stimulatory” responses, may preserve key immunoregulatory cells and give the host time to mount effective immune responses. Regardless, these findings may hold promise for design of novel preventive and therapeutic strategies to combat HIV infection.
